# DNA Damage Response and Repair in Boron Neutron Capture Therapy

**DOI:** 10.3390/genes14010127

**Published:** 2023-01-02

**Authors:** Grigory V. Mechetin, Dmitry O. Zharkov

**Affiliations:** 1 Department of Natural Sciences, Novosibirsk State University, 2 Pirogova St., 630090 Novosibirsk, Russia; 2 Siberian Branch of the Russian Academy of Sciences Institute of Chemical Biology and Fundamental Medicine, 8 Lavrentieva Ave., 630090 Novosibirsk, Russia

**Keywords:** boron neutron capture therapy, high-LET radiation, DNA damage, DNA repair, double-strand breaks, clustered DNA lesions, homology-directed recombination, non-homologous end joining, base excision repair

## Abstract

Boron neutron capture therapy (BNCT) is an approach to the radiotherapy of solid tumors that was first outlined in the 1930s but has attracted considerable attention recently with the advent of a new generation of neutron sources. In BNCT, tumor cells accumulate ^10^B atoms that react with epithermal neutrons, producing energetic α particles and ^7^Li atoms that damage the cell’s genome. The damage inflicted by BNCT appears not to be easily repairable and is thus lethal for the cell; however, the molecular events underlying the action of BNCT remain largely unaddressed. In this review, the chemistry of DNA damage during BNCT is outlined, the major mechanisms of DNA break sensing and repair are summarized, and the specifics of the repair of BNCT-induced DNA lesions are discussed.

## 1. Introduction

Radiation cancer therapy is a widely used alternative or supplement to the surgical removal of localized solid tumors and is also routinely combined with chemotherapy [1]. Generally, tumors are irradiated using either high-energy photons (X-rays or γ-rays) or accelerated particles (protons, neutrons, or carbon ions). Side effects in normal tissues in the way of the beam are common, encouraging a search for regimes that would maximize the sensitivity of tumor cells and allow the use of lower radiation doses. One such method was proposed in 1936, soon after the discovery of a new subatomic particle, the neutron, and nuclear reactions involving it [2]. Neutrons are very efficiently captured by the nuclei of a stable boron isotope, ^10^B, which then decays by α particle emission. If there was a way to concentrate ^10^B in tumor cells, they would be selectively exposed to radiation, while the surrounding tissues would be spared because, unlike neutrons, α particles can penetrate tissues to very shallow depths. Moreover, due to the large cross section of the ^10^B reaction, the energy of incoming neutrons may be low (epithermal neutrons), decreasing the damage from the primary radiation. Thus, the concept of boron neutron capture (BNC) therapy (BNCT) was born.

While conceptually simple, two technical hurdles severely restricted the practical application of BNCT, namely the lack of good boron carriers delivering ^10^B into cells and the lack of compact and safe neutron sources. Over the past 20 years, significant progress was made in both fields, and BNCT is now in clinical use in the US, Japan, China, Russia, and several other countries with operational reactors or, more recently, accelerator neutron sources [3,4,5]. Historically, BNCT attracted significant interest as a therapy for aggressive diffuse brain tumors such as glioblastoma multiforme [6,7] (Table 1). However, a number of clinical studies have now addressed, albeit at a smaller scale, the application of BNCT in other forms of cancer (Table 1). Several dedicated models of dose–rate–effect relationships have been developed to take into account the specifics of BNCT, such as the long exposure and the spatial and temporal inhomogeneity of the boron distribution [8,9,10]. Several excellent recent reviews covered the basic principles and clinical applications of BNCT [11,12,13].

What lags behind the technical advances and clinical studies is an understanding of the essentials of BNCT at the molecular level. Mechanistic studies of events in the cell following BNC are very limited, and most of today’s considerations on this topic are implicitly based on information about the biological effects of high linear energy transfer (high-LET) radiation from other sources and radiomimetic drugs. Thus, it is important to understand to what extent BNC-induced damage and the cell’s reaction to it are similar to other kinds of radiation damage.

## 2. DNA Damage: The Essence of Radiation-Induced Cell Death

The ultimate cause of cell death after exposure to a lethal dose of ionizing radiation is DNA damage. In normal cells that maintain their proliferative potential (e.g., stem cells), the damage levels that overload the repair capacity activate cellular mechanisms of apoptosis (or, in some cases, other cell death pathways). Cells possess a tightly regulated network of DNA damage response that senses various kinds of lesions in DNA, triggers DNA repair, and accordingly regulates the cell cycle to allow time for repair [26,27,28,29]. In cancer cells, which also proliferate constantly, some of these mechanisms are dysfunctional, and a cell with its DNA damaged may persist for some time with pronounced genome instability and rearrangements; however, cancer cells with massive genome damage also succumb to cell death in one or another way [30,31,32]. Non-dividing terminally differentiated cells, such as adult neurons, are more resistant to DNA damage, but accumulating lesions also disturb their functions, ultimately proving lethal [33,34,35,36].

The nuclear reaction underlying BNCT is:

^10^B + *n* → ^7^Li + ^4^He

**Figure 1:**
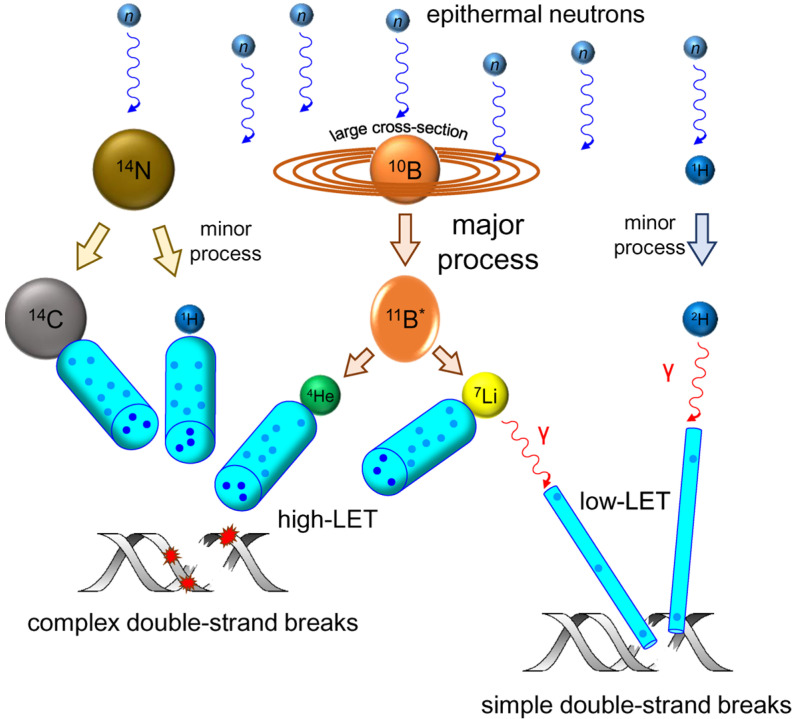
Schematic depiction of nuclear reactions, ionization processes, and DNA damage taking place during BNCT.

The main specific sources of damage are high-LET α particles (^4^He nuclei) and Li^+^ ions. Both products of this nuclear reaction have close LET values and energies at the same order of magnitude (~150 keV/μm and 1.47 MeV for the α particle and ~175 keV/μm and 0.84 MeV for Li^+^) and thus produce densely ionized tracks that are limited in length to ≤10 μm [37] (Figure 1). One primary ionization event takes ~100 eV of energy [38], so around 1500–2000 such events may be packed into a 1 µm path length. The major primary products of radiolysis in aqueous media are electronically excited water (H_2_O*), water radical cations (H_2_O^•+^), and electrons (*ē*), which all immediately react with other water molecules to yield hydroxyl radicals (HO^•^), monoatomic hydrogen radicals (H^•^), and solvated electrons (*ē*_aq_) as well as ubiquitously present H^+^ and OH^−^ ions. Further recombination produces other reactive oxygen species such as hydrogen peroxide (H_2_O_2_) and superoxide anion radicals (^•^O_2_^−^), which become more abundant if molecular oxygen is present in the solution, as it is in living cells. Additionally, most of the excited ^7^Li nuclei relax by emitting low-LET γ photons, producing sparsely ionized tracks of the same reactive species. Besides ^10^B atoms, epithermal neutrons can be much less efficiently captured by ^1^H (^1^H + *n* → ^2^H + γ) or ^14^N (^14^N + *n* → ^14^C + ^1^H). Due to the sheer amount of ^1^H and ^14^N in the cell, this produces some background low-LET (γ photons) and high-LET (charged ^14^C nuclei and protons) radiation independent of the presence of boron carriers. As a result of these modes of neutron interaction with matter, BNC exposes cells to mixed-field radiation, in which high-LET and low-LET particles yield lesions that are chemically similar but differently distributed [39,40] (Figure 1). It is possible that the complex nature of mixed field radiation induced damage is at least partly responsible for the unwanted effects observed in clinical settings, such as the necrosis of surrounding normal tissue and tumor pseudoprogression (an increase in the tumor volume due to immune cell infiltration) [13].

**Figure 2:**
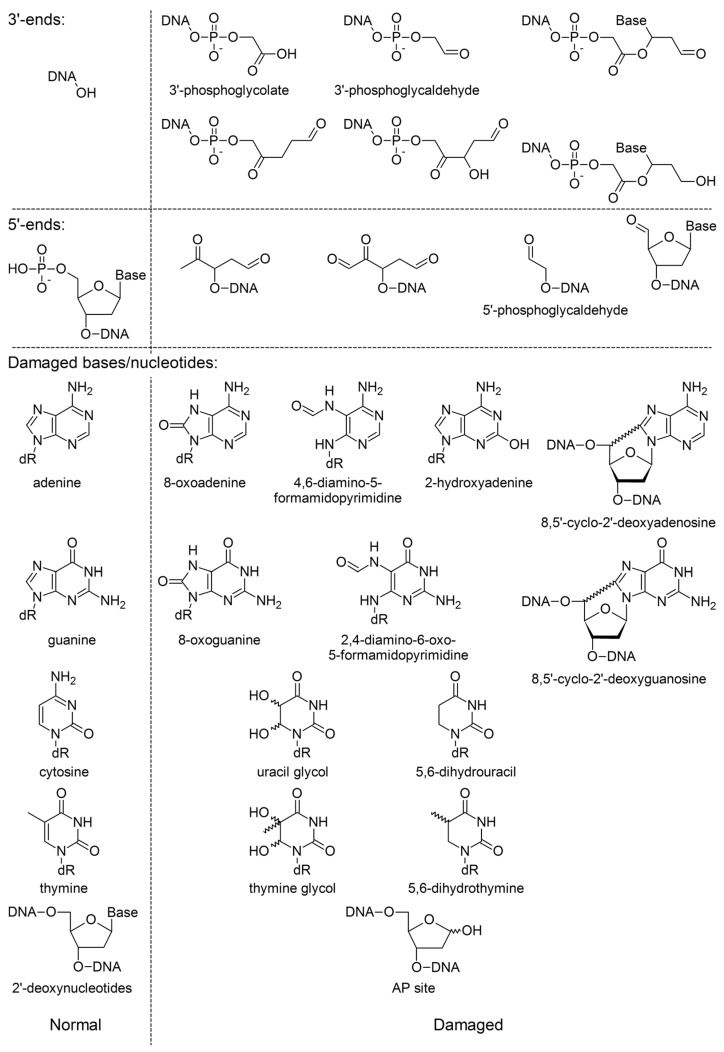
Structures of selected ionizing-radiation-generated DNA break ends, damaged bases, and nucleotides and their parent moieties. “Normal” 3′ ends are those extendable by DNA polymerases without additional enzymatic processing. “Normal” 5′ ends are those that can be ligated without additional enzymatic processing. In “Damaged bases/nucleotides”, for the sake of brevity, 2′-deoxyribose is shown as dR, and base rather than nucleotide names are given if only the base is damaged.

When tracks of reactive water-derived species cross the cell’s nucleus, the radicals react with DNA in multiple ways. Given that the average distance between the primary ionization events is <1 nm and the characteristic dimensions of B-DNA are ~2.0 nm diameter and 3.32 nm per helix turn, it is almost guaranteed that high-LET radiation will generate several lesions spaced very closely in DNA, possibly within a single helix turn [41,42]. Often, the reactions involve the sugar–phosphate DNA backbone, which usually leads to a DNA break (Figure 2). The C5 and C6 positions in pyrimidine nucleobases and the C4, C5, and C8 positions in purines are also susceptible to the attack, producing a variety of damaged bases (Figure 2). Damaged bases can be also lost from DNA, generating baseless deoxyribose, commonly known as apurinic/apyrimidinic (AP) sites. The spectrum of these products may vary considerably, depending on the availability of dissolved O_2_. However, in many cases the lesions involve two single-strand DNA breaks (SSBs) spaced closely enough to produce a double-strand break (DSB).

At present, the specifics of BNC-produced DNA damage seem to be fairly similar to other types of high-LET damage [43]. A convenient way to study BNC-induced reactions is the neutron irradiation of aqueous solutions containing boron compounds (boric acid in the simplest variant) and an appropriate target such as plasmid DNA [44,45,46]. Such model systems produce estimates of the SSBs and DSBs expected for the high-LET particles of the respective energy. Interestingly, some clinically used boron carriers, such as borocaptates, act as free radical scavengers, decreasing the yield of strand breaks [47].

Thus far, two boron carriers used in clinical settings are boronophenylalanine and sodium borocaptate, which are distributed within cells in a stochastic manner [48,49]. However, these are far from being selective for cancer cells, and their uptake varies significantly from one cell type to another, introducing considerable unpredictability into boron delivery to the tumor [50,51]. Given that DNA is the ultimate target for the lethal damage and that, in brain tumors, cancer cells replicate much faster than normal cells, many attempts have been made to develop boron carriers that deliver ^10^B directly to the genome [52]. Such carriers include boronated nucleosides and nucleotides [53,54,55,56,57], DNA intercalators [58] and DNA-binding peptides [46,59]. Despite some success in in vitro experiments, so far none of these carriers have made it into clinical trials.

## 3. Break Detection and Repair Pathway Choice

DSBs are among the most lethal kinds of genome lesions. Besides ionizing radiation, sources of DSBs include many chemical agents [26,38,60,61] and intrinsic processes, including DNA cleavage by topoisomerases [62] and specialized nucleases [63], replication over damaged DNA [64], and the repair of closely spaced damaged nucleotides [65]. To repair such potentially lethal damage, two main protective mechanisms have evolved: homology-directed recombination (HDR) and non-homologous end joining (NHEJ). HDR engages an intact copy of the genetic material (a sister chromatid or a homologous chromosome; in human cells, sister chromatids are overwhelmingly used [66]) to restore the broken copy in an error-free manner. NHEJ, on the other hand, seals the break with no external template at the expense of losing some DNA surrounding the lesion. Hereditary deficiencies in human HDR and NHEJ systems cause rare but grave disorders such as ataxia telangiectasia (Louis–Bar syndrome), severe combined immunodeficiency, and Nijmegen breakage syndrome [26,67,68].

Since the sister chromatid is available as a template in dividing human cells only for a short interval during the cell cycle, HDR mainly operates in the late S phase and at the S/G2 border, whereas outside of this time window DSB repair in eukaryotic cells proceeds mainly through NHEJ [69]. Moreover, the main NHEJ factor, the Ku heterodimer (see Section 5), is found in high amounts in human cells and has high affinity for DSBs, so NHEJ kinetically outperforms HDR, which occurs when the repair is delayed due to the modification of DSB ends or high chromatin condensation in the region of the DSB [70].

### 3.1. DSB Sensing

**Figure 3:**
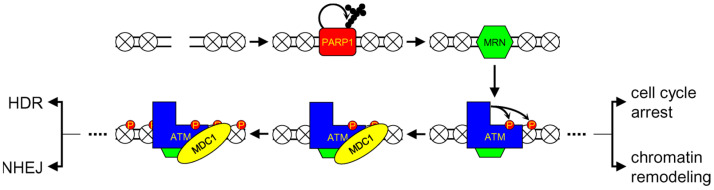
Scheme of initial events during DSB sensing. PARP1 senses the damage, self-modifies, and recruits MRN. MRN in turn attracts ATM, which phosphorylates (red circles) itself and H2AX in the surrounding nucleosomes (crossed circles). MDC1 binding helps spread H2AX phosphorylation over the adjacent regions of chromatin.

The general sequence of events after a chromosomal break in eukaryotic cells was largely established through studies of the genetics and biochemistry of unicellular eukaryotes, mostly baker’s yeast (*Saccharomyces cerevisiae*) and fission yeast (*Schizosaccaromyces pombe*), and subsequently refined and supplemented by data from cultured human and rodent cells. At present, the scheme in human cells appears as follows: The primary damage sensor is poly(ADP-ribose) polymerase 1 (PARP1), which was long believed to be a central SSB sensor but has now also been implicated in early DSB recognition. PARP1 binds to a DSB, undergoes automodification by poly(ADP-ribose) (PAR) chains (PARylation), and serves as a signal attracting the MRN complex (MRE11/RAD50/NBS1, equivalent to Mre11p/Rad50p/Xrs2p in *S. cereviseae* (MRX)), the key factor in DSB recognition and HDR initiation (Figure 3) [71,72,73,74,75]. MRN then binds and activates ATM protein kinase [76,77,78]. ATM phosphorylates itself and histone H2AX [77,79], producing H2AX-phosphoSer139 (γH2AX), which binds the mediator of DNA damage checkpoint protein 1 (MDC1) factor [80,81]. MDC1 has a high affinity for MRN, which triggers the binding of additional MRN and ATM molecules near the initial DSB and the spreading of γH2AX for tens to hundreds of thousands of base pairs, with great signal amplification [81,82]. With appropriate immunostaining (e.g., by anti-γH2AX antibodies), the protein complexes formed in the DSB regions can be microscopically observed and are sometimes called ionizing-radiation-induced foci (IRIFs). IRIF formation can occur, even in the absence of ATM, in which case H2AX is phosphorylated by another protein kinase, ATR, but the efficiency of this process is much lower [83].

As central factors in cell cycle checkpoints, ATM and ATR trigger a signaling cascade that arrests the cell cycle in the presence of DSBs, allowing time for repair via the HDR or NHEJ pathways [84]. Among the targets of ATM/ATR are CHK1 and CHK2, the master kinases that regulate progress through the G1/S and G2/M checkpoints [77,85,86,87]. MDC1 recruits ubiquitin protein ligases RNF8 and RNF168 to IRIF, which ubiquitylates histone H2A [88,89,90]. This form of histone H2A also extends tens of thousands of base pairs from the DSB. The ubiquitylation of H2A induces the histone-specific methyltransferase-MMSET-dependent deposition of H4K20me2 [91]. It has been suggested that by binding near DSBs, RNF8 and RNF168 also ubiquitylate Jumonji-domain-containing (JMJD) lysine demethylases that compete with MMSET, which leads to the proteolytic destruction of JMJDs [92]. The 53BP1 factor binds to H2AK20me3 [91], which causes further signal amplification due to the 53BP1 affinity for MRN. The ubiquitylation of H2A by RNF8 presumably causes the binding of the RAP80/BRCC36/ABRAXAS complex, which stimulates the loading of BRCA1 to the damage site [93,94]. IRIFs also contain the sumoylation factors UBC9, PIAS1, and PIAS4, which are required for DSB binding by 53BP1 and BRCA1 [95], and 53BP1 and RAP80/BRCC36/ABRAXAS protect the ends of the DSB from the resection by HDR pathway enzymes (see Section 4), allowing the Ku heterodimer to attract NHEJ proteins. If, however, NHEJ is delayed and the cell enters the S/G2 phase of the cell cycle, BRCA1 expels 53BP1 and RAP80/BRCC36/ABRAXAS from IRIFs, exposing the DSB ends to the enzymes of the HDR system [96,97,98]. BRCA1, RAD51, and RAD54 (see below), but not Ku, are upregulated after BNCT in hepatocellular and thyroid carcinoma cells, suggesting that HDR is the primary repair pathway for BNC-induced damage [99,100,101].

Recently, the role of non-coding RNAs (ncRNAs) in the regulation of the detection and initiation of DSB repair has been shown [102]. The downregulation of the DICER and DROSHA RNases involved in the formation of ncRNA leads to a significant suppression of IRIF formation and ATM autophosphorylation, decreasing the activity of this protein kinase and preventing cell cycle arrest at the G1/S and G2/M checkpoints, which is necessary for DSB repair. Although the regulation of DSB repair by ncRNAs seems to be important, its mechanism remains to be established [102,103].

Extensive DNA damage that overwhelms the cell’s repair capacity triggers several kinds of cell death as well as autophagy and cell senescence. Several recent reviews comprehensively covered these topics with respect to high-LET-induced damage [30,104,105].

### 3.2. Chromatin Remodeling

In human cells, a significant fraction of the genomic DNA exists in the form of highly compacted chromatin, which hinders the access of the HDR and NHEJ factors to DSBs. Several mechanisms of chromatin remodeling operate to provide access for the repair proteins to the DSB and the adjacent DNA. On the other hand, active transcription in the region of a DSB impedes its repair, and signaling pathways exist that compact euchromatin and suppress transcription immediately before the start of repair.

In euchromatin, the DSB-associated PARP1 PARylates the nucleosome and/or associated proteins, attracting the NuRD and ALC1 chromatin remodeling complexes as well as the KAP1 (TRIM28) and HP1 (CBX5) factors to the damage site, which leads to short-term chromatin compaction [106,107,108,109,110]. If chromatin decompaction is required, the MDC1 protein accumulating in the DSB region causes the binding of the TIP60 chromatin remodeling complex, which contains the Domino/p400 histone chaperone that replaces H2A with the H2A.Z variant, promoting H4 acetylation by another NuA4 component, TIP60 histone acetylase [111]. The acetylation of H4 and the destabilization of H2A.Z-bearing nucleosomes cause a decrease in the chromatin packing density, allowing signal amplification by the DSB detection system and providing the necessary environment for further break repair along one of the pathways. Interestingly, histone deacetylase inhibitors such as valproic acid sensitize cells rather than protecting them from BNCT [112]. This may seem counterintuitive, but it is likely related to the increased solvation of decompacted DNA enhancing its damage. It should be noted, however, that under some conditions (such as the presence of other chromatin modifications or other histone variants) H2A.Z, on the contrary, can stabilize nucleosomes [113,114]. Upon H2A.Z depletion, DSB ends undergo uncontrolled degradation, which can be rescued by a loss of CtIP, indicating a possible role of H2A.Z in controlling the depth of the hydrolysis of the DSB ends by the MRN-CtIP complex [115,116].

In heterochromatin, DSB-activated ATM kinase phosphorylates KAP1, forcing NuRD to leave the chromatin [117] and thus causing a decrease in heterochromatin density and further relaxation involving TIP60 [118].

## 4. Homology-Directed Recombination

**Figure 4:**
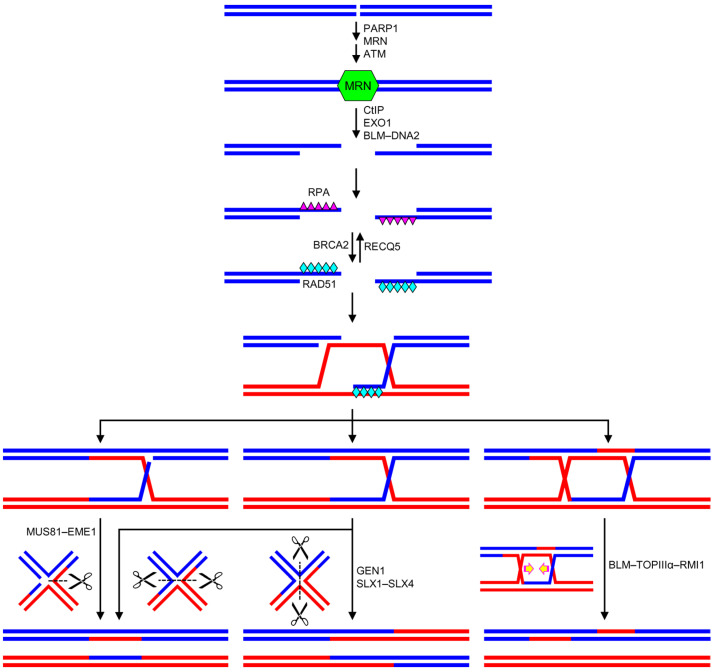
Principal scheme of homology-directed recombination in human cells. MRN is recruited and activated in a PARP1- and ATM-dependent manner (Figure 3). Next, CtIP, EXO1, and BLM–DNA2 trim the break ends, forming recombinogenic 3′-terminal single-strand tails, which are quickly covered by RPA. BRCA2 induces the exchange of RPA for RAD51 and strand invasion. After DNA synthesis and ligation, Holliday junctions are resolved by GEN1, SLX1–SLX4 (regular junctions), MUS81–EME1 (junctions with a nick), or BLM–TOPIIIα–RMI1 (fusing two junctions).

HDR in human cells begins with the formation of overhanging 3′ ends. The MRN trimer recruited to the DSB binds the CtIP endonuclease; the MRN–CtIP complex trims the DNA at the break, forming the 3′-protruding ends [115]. It is assumed that the resection is initiated by an incision at some distance from the DSB by the endonuclease activity of MRN–CtIP and then proceeds towards the break due to the CtIP-activated 3′ → 5′ exonuclease activity of MRE11 (Figure 4) [119]. A loss of CtIP shifts the repair towards alternative end-joining or strand annealing pathways and greatly increases the yield of chromosome abnormalities after high-LET irradiation [120]. The protruding 3′ ends are further extended by the 5′ → 3′ exonuclease EXO1 or the helicase–5′ → 3′ exonuclease BLM–DNA2 complex [119,121,122,123]. As the single-stranded tails become exposed, they are covered by the RPA protein to protect them from accidental destruction. RPA-associated single-stranded DNA binds the ATRIP protein and ATR kinase, activating cell cycle arrest signaling pathways [124,125,126]. The BRCA2 factor swaps RPA for the RAD51 strand exchange protein, resulting in the ATP-dependent formation of a heteroduplex with a sister chromatid or a homologous chromosome and the formation of the Holliday structure [127,128]. The formation of the nucleoprotein filament is negatively regulated by the RECQ5 helicase, which removes RAD51 from DNA. Mutations that inactivate RECQ5 lead to hyper-recombination, chromosome rearrangements, and cancer predisposition [129,130]. The RAD54 helicase binds to the DNA/RAD51 nucleoprotein filament and destabilizes polymerized RAD51, allowing DNA polymerases to extend from the invading 3′-terminus and promote further branch migration. Interestingly, this factor can both stabilize the Holliday structure and lead to its disruption. It remains unclear how these two activities lead to the productive migration of the junction [131].

At the final stage, the Holliday structure is resolved, which can be carried out in several ways (Figure 4). First, the GEN1 nuclease or the SLX1–SLX4 complex resolves the complete Holliday structure [132,133,134]. Alternatively, the MUS81–EME1 complex effectively resolves the Holliday structure in the presence of a break, cleaving the second strand at the point opposite the break [135]. Finally, the BLM–TOPIIIα–RMI1 complex, which has helicase and topoisomerase activities, can fuse two closely spaced crossovers, dissolving them without DNA cleavage [136,137].

Finally, the repair may proceed through the synthesis-dependent strand annealing pathway. The processing of the 3′-overhanging ends in this case depends on the ERCC1-XPF endonuclease [138,139]. After the strand invasion and extension, the FANCM factor causes the dissociation of the Holliday structure [140,141]. Damaged DNA is repaired by the re-annealing of the nascent strand with the remaining 3′-overhanging end, followed by gap filling and ligation.

## 5. Non-Homologous End Joining

**Figure 5:**
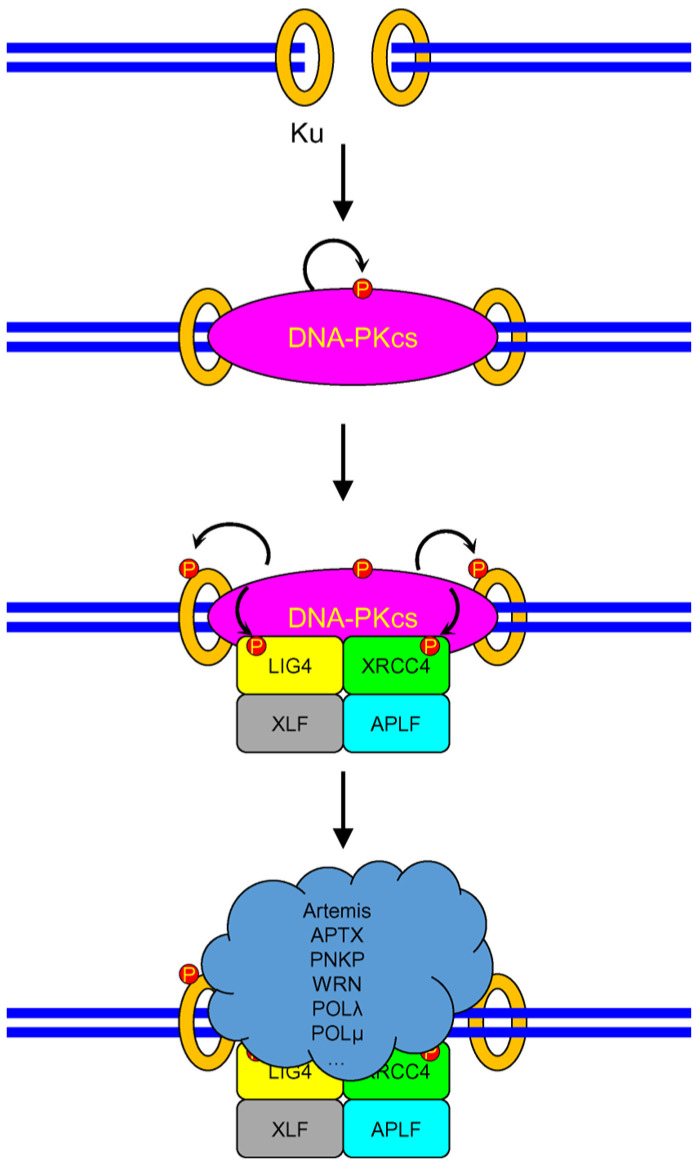
Principal scheme of non-homologous end joining in human cells. DSB ends are recognized by Ku, followed by DNA-PKcs binding and self-phosphorylation (red circles). This promotes the binding of LIG4/XRCC4, XLF, and APLF. After Ku and LIG4/XRCC4 phosphorylation, DNA-PKcs releases the DSB ends and allows the assembly of the rest of the NHEJ machinery.

The central place in the NHEJ pathway of DSB repair is occupied by the heterodimeric Ku70/Ku80 protein. Due to its high affinity for the DSB ends and high abundance in the cell [142], the Ku70-Ku80 heterodimer quickly binds DSBs, thereby protecting their ends from hydrolysis (Figure 5). Structurally, the Ku heterodimer is doughnut-shaped, resembling DNA polymerase processivity factors (bacterial DnaN and eukaryotic PCNA and 9-1-1 complexes) [143]. Then, the catalytic domain of DNA-dependent protein kinase (DNA-PKcs) binds to the Ku70/Ku80–DSB complex, after which Ku70-Ku80 retreats from the break along the DNA strand by about one DNA turn [144], allowing the protein kinase to bind the DSB end directly. DNA-PKcs in complex with a DSB forms a catalytically active protein kinase (DNA-PK). The key event of the NHEJ pathway is DNA-PK autophosphorylation, which stabilizes the complex and promotes the binding of the subsequent participants in the process: the LIG4–XRCC4 complex and XLF and APLF factors. DNA-PK phosphorylates XRCC4, LIG4, and Ku70 and leaves the complex, exposing the ends for repair [145].

Since DSBs can be caused by a variety of factors, the chemical nature of DSB ends can also vary considerably. A set of specific enzymes serves to remove these modifications and clean up the ends. XRCC4 assembles a repair complex consisting of aprataxin (APTX) and polynucleotide kinase-3′-phosphatase (PNKP) [146,147,148], while the Artemis factor is engaged through interactions with LIG4 and DNA-PK [149,150,151] (Figure 5). APTX possesses 5′-AMP-DNA hydrolase activity and removes 5′-AMP resulting from DNA ligase errors that cause DSBs [152]. PNKP removes the 3′-phosphate and phosphorylates the 5′ ends of DSBs, preparing them for rejoining, while Artemis can remove 3′-phosphoglycolate [153,154]. The Ku heterodimer has AP lyase/deoxyribophosphatase activity and is able to remove AP sites from the 5′-termini of DSBs [155]. The repair complex also contains WRN DNA helicase, which binds to DSBs by interacting with the Ku heterodimer and can remove modifications from the 3′ ends of a DSB due to its 3′ → 5′-exonuclease activity [156,157,158]. Some enzymes that may participate in break processing before rejoining may work either fully or partly independently of the NHEJ repair complex. For example, covalent adducts of DSBs with topoisomerases are cleaved by tyrosyl-DNA-phosphodiesterases 1 and 2 (TDP1 and TDP2), and 3′-phosphoglycolate can be removed by AP endonuclease 1 (APEX1) [159] and TDP1 [160,161,162] without the need for prior break recognition by DNA-PK. Nevertheless, Artemis and TDP1 appear to cooperate in the removal of 3′-phosphoglycolate from most DSBs in NHEJ [163].

To complete the repair, the gaps at the ends of the DSBs are filled by DNA polymerases μ or λ [164]. The polymerases are assembled into a repair complex by interacting with the Ku heterodimer; however, LIG4 and XRCC4 are required for their stable work in the complex, and XLF stimulates the filling of mutually non-complementary gaps in the final stage of NHEJ. Then, the gap is ligated by the LIG4–XRCC4 complex, stimulated by XLF and APLF, and the complex dissociates. The Ku heterodimer remains encircling the DNA and must be degraded through polyubiquitylation by the SCF ubiquitin ligase complex and proteolysis [165,166].

Besides the classical NHEJ pathway, two other error-prone pathways have gained considerable attention in recent years [167]. The “alternative NHEJ” (Alt-NHEJ, also known as microhomology-mediated end joining (MMEJ)) pathway begins essentially as HDR, with PARP1-mediated MRN recruitment and CtIP-dependent end resection [168]. However, if a short stretch of complementarity (2–20 nt) occurs in the exposed single-stranded tails, they can anneal to each other. The non-complementary flaps are removed by ERCC1–XPF endonuclease, the gaps are filled in by DNA polymerase θ, and the LIG3α/XRCC1 complex seals the last nick [169,170]. Alternatively, if the break is located in a highly repetitive region, the 3′-tails may be further extended by EXO1 or BLM–DNA2 and annealed over >20 nt by RAD52 in a so-called single-strand annealing pathway [167]. Following that, flap processing, gap filling, and ligation likely proceed as in alt-NHEJ.

## 6. Clustered Non-DSB Lesions and Base Excision Repair

**Figure 6:**
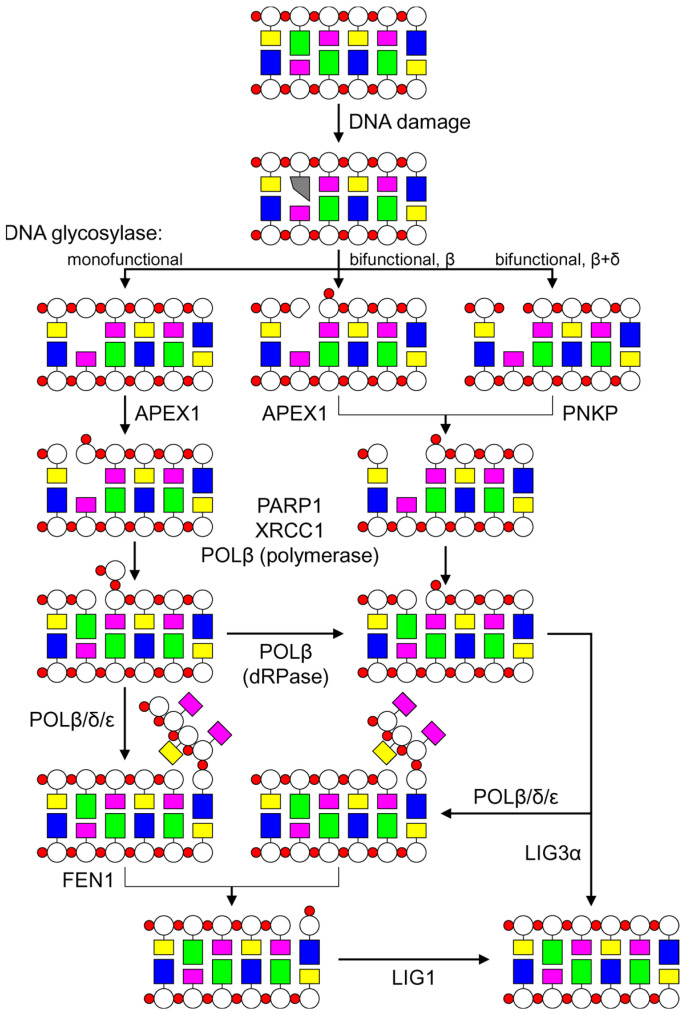
Principal scheme of base excision repair in human cells. Colored rectangles represent nucleobases; white circles, 2′-deoxyribose; red circles, internucleoside phosphate groups. After DNA damage, the damaged base is excised by a DNA glycosylase. Depending on the nature of the product of this reaction, the 3′ end is cleaned by APEX or PNKP. Extension by POLβ can be channeled into either a short-patch (POLβ → LIG3α) or long-patch (POLβ/δ/ε → FEN1 → LIG1) subpathway.

Radiation-induced lesions other than DSBs are generally repaired via the base excision repair (BER) pathway. Despite its name, BER deals not only with damaged nucleobases but also with AP sites and SSBs. If a base is damaged, BER is initiated by the cleavage of its *N*-glycosidic bond by one of the enzymes that belong to the class of DNA glycosylases [171] (Figure 6). DNA glycosylases are divided into several types according to their mechanisms of action and the products they generate. Monofunctional DNA glycosylases excise the target base and leave an intermediate repair product, the AP site. As mentioned above, AP sites can also arise due to spontaneous base loss, including that triggered by ionizing radiation. Other DNA glycosylases are classified as bifunctional since after the removal of the damaged base they introduce a break at the 3′ side of the nascent AP site. Because of this, some DNA glycosylases were erroneously called endonucleases upon discovery (e.g., *E. coli* endonucleases III and VIII and their human homologs NTHL1 and NEIL1–NEIL3) and remain known by these names, although they cleave the DNA backbone not by hydrolysis but by the elimination of the 3′-phosphate group (β-elimination) and thus are not endonucleases but rather lyases in terms of the reaction mechanism [172,173]. The products of β-elimination cannot serve as substrates for DNA polymerases and must be removed from DNA. Some bifunctional DNA glycosylases are capable of catalyzing two successive reactions of the elimination of 3′- and then 5′-internucleoside phosphates (β,δ-elimination), thus converting the lesion into a single-nucleoside gap flanked by two phosphates, which must also be processed prior to repair DNA synthesis. These differences in the reaction mechanism lead to the separation of BER into several subpathways. AP sites produced by monofunctional DNA glycosylases serve as substrates for APEX1 AP endonuclease, which hydrolyzes the phosphodiester bond immediately 5′ of the AP site and thus introduce an SSB into DNA [174] (Figure 6). The 3′-unsaturated sugars produced by β-elimination are removed from DNA by phosphodiesterase activity, which is usually also associated with APEX1 [174]. The 3′-phosphate groups arising after β,δ-elimination are cleaved from DNA by phosphatase activity, which in human cells belongs to polynucleotide kinase/3′-phosphatase (PNKP) [175]. Finally, in the nucleotide incision repair (NIR) subpathway, AP endonucleases cleave DNA 5′ of some dNMPs still bearing damaged bases without their prior removal by a DNA glycosylase [176].

The hydrolysis of DNA at the AP site by AP endonucleases leads to the appearance of another BER intermediate, an SSB flanked by a 3′-terminal OH group serving as a substrate for DNA polymerases, and a 5′-terminal 2′-deoxyribose-5′-phosphate (dRP) fragment. To complete the repair, gap-filling DNA synthesis and subsequent nick ligation are required. The SSB is sensed by PARP1, which loads the XRCC1 scaffold protein; in turn, XRCC1 binds and correctly positions DNA polymerase β (POLβ) and DNA ligase IIIα (LIG3α). At this stage, BER again splits into two subpathways called short-patch and long-patch BER (Figure 6). In the former, POLβ incorporates a single undamaged dNMP. The dRP fragment is removed from DNA by a 2′-deoxyribose-5′-phosphate lyase activity, which in human cells mainly resides in the N-terminal domain of POLβ. The dRP removal clears the way for ligation by the LIG3α/XRCC1 complex. In long-patch BER, after the incorporation of the first dNMP, the repair synthesis continues with the displacement of several nucleotides as a flap. This step may also be carried out by POLβ or involve DNA polymerases δ or ε, together with their auxiliary factors (PCNA, RFC, and RPA). The displaced flap is cut off by FEN1 flap endonuclease, and the single-strand break is ligated, in this case, by DNA ligase I [177].

Unless a radiation track produces two closely spaced breaks in the opposite strands, the resulting damage is not a DSB but is rather classified as a “clustered lesion”, which may contain a break and a damaged base or two (or more) damaged bases or AP sites. Damaged sugars are usually quickly converted into breaks [178] and therefore are rarely considered as a separate class of lesions, at least as their consequences are concerned. Within clustered lesions, a subclass of “tandem lesions” formed by two damaged units in one strand is less dangerous since one DNA strand maintains its integrity and can serve as a template to repair both lesions. Bi-stranded lesions, on the other hand, either require tight coordination of the repair steps to avoid a DSB formation or may have to be controllably converted to a DSB that is then repaired.

Tandem lesions are generally repaired by BER with little difficulty. However, if the damaged units are in the adjacent positions (“vicinal tandem lesions”) they may be poorly recognized by DNA glycosylases and AP endonucleases, the enzymes that initiate BER, either due to the non-canonical DNA structure under the protein’s footprint or because the cleavage at one site inhibits the action of the enzyme at the second site [179]. Ionizing radiation has been reported to produce a significant fraction of such refractory tandem lesions [180]. Even if two lesions in a tandem are separated by a few nucleotides, they may affect each other’s recognition by BER enzymes [181,182,183]. In such situations, if the first cleavage occurs at the 3′-terminal lesion, further repair is often suppressed, while the initial cleavage at the 5′-terminal lesion is permissive of the repair of the second lesion [181]. In the latter case, the repair may proceed through the long-patch BER pathway with the displacement of the strand carrying the 3′-terminal lesion, followed by the action of FEN1 [184]. The only type of radiation-induced damage that is predominantly repaired not by BER but by nucleotide excision repair is 8,5′-cyclopurines, which are also classified as tandem lesions despite technically being produced from a single nucleotide [185,186].

The processing of closely spaced bi-stranded lesions strongly depends on their arrangement. For example, two AP sites positioned 3′ to each other are efficiently converted to a DSB by APEX1, while in the opposite arrangement the cleavage is either blocked or limited to one strand, depending on the distance between the AP sites [187,188,189,190]. Nuclear extracts contain activities enhancing clustered AP site cleavage by APEX1 in the context of a nucleosome, but they remain to be identified [191], and nucleosomes themselves promote the conversion of AP site clusters to DSBs [192,193]. AP sites or SSBs in the opposite strand to 8-oxoguanine inhibit the excision of this base by human OGG1 DNA glycosylase [189,194,195], which should give the repair priority to the AP- or SSB-containing strand and help avoid a DSB. Three-lesion clusters (two tandem or bi-stranded base lesions plus an AP site) present a significant delay in the repair [196,197,198]. Interestingly, radiation-induced clustered AP sites and oxidized bases persist in human and rodent cells of different origins for up to an order of magnitude longer than DSBs, suggesting a possible lack of a dedicated mechanism to resolve this kind of damage [199,200]. DNA-PKcs and BRCA1 were reported to facilitate the repair of radiation-induced non-DSB clustered lesions in living cells, while Ku inhibits several oxidized-base-specific DNA glycosylases, supporting the controllable DSB formation hypothesis [201,202,203].

## 7. Relevance to BNCT

Compared with low-LET, high-LET-induced damage requires significantly more time for repair, as evidenced by the IRIF and RAD51 foci disappearance kinetics [204]. In BNCT, γH2AX and 53BP1 IRIFs persist for much longer than those induced by γ-irradiation, although the absolute level of foci is markedly cell-type-specific [100,205,206,207]. IRIFs are also larger and more tightly clustered in BNCT-treated cells, which may be suggestive or a more complex character of DNA damage [100,208]. The PAR formation dynamics in a rat tumor graft model also suggest delayed cellular response to BNC-produced damage [209].

The poor repairability of DSBs caused by high-LET radiation, and BNCT in particular, raises questions related to the reasons underlying this phenomenon. One possibility may be the complex chemistry of the ends of such DSBs. Damaged bases and AP sites are often found in their vicinity, and the ends have been shown to carry 3′-OH, 3′-phosphate, and 5′-phosphate groups but relatively low amounts of 3′-phosphoglycolate, a characteristic product of low-LET radiation [210,211,212]. While these terminal modifications of a DSB are unlikely to affect the end processing, damaged bases and AP sites might do so. For example, AP sites, 8-oxoguanine, and 8-oxoadenine block the 3′ → 5′-exonuclease activity of WRN, especially in the absence of the Ku complex [213,214]. In addition, the slow processing of non-DSB clustered lesions may also contribute to the delayed repair of BNCT-induced damage. Moreover, recall that BNCT produces mixed-field rather than purely high-LET radiation; it is possible that the simple DSBs induced by the low-LET component can divert the repair machinery for their immediate repair, giving complex DSBs less priority.

Another model is based on the prevalence of clustered DSBs in the spectrum of high-LET-induced lesions. For reasons that are not fully understood, NHEJ is suppressed in clustered DSBs [120,215]. In several human cancer cell lines, BNCT-induced lesions are repaired mostly through HDR [99,100,101], although in melanoma NHEJ may also contribute [100]. On the other hand, observations in rodent cells (mouse embryonic fibroblasts and Chinese hamster fibroblasts and ovary cells) indicate that the repair of BNC-induced damage partly depends on LIG4, Ku80, and DNA-PKcs, suggesting that, at least in these cell types or perhaps in rodents in general, NHEJ contributes to the protection against complex-end DSBs [205,216,217,218]. PARP1 inhibitors apparently do not potentiate the lethal effect of BNCT, although these studies were also conducted in rodent cells [217].

Overall, BNCT appears to be the safest way to deliver high-LET ionizing radiation to cancer cells and induce complex lesions in their DNA that are not easily repairable. Intracranial tumors, predominantly gliomas, account for most attempts to use BNCT in clinical settings, using the terminally differentiated nature of neurons to the advantage of a therapy based on lethal-upon-replication genome damage. The current main challenges in the field seem to be the absence of specific boron carriers that can selectively target tumor cells as well as a lack of understanding of the complex effects of mixed-field radiation, causing unwanted side effects. In the future, BNCT may also benefit from the development of drugs that specifically suppress HDR [219,220], while targeting BER requires further investigation.

## Figures and Tables

**Table 1:** Selected clinical studies of BNCT in various types of cancer.

Cancer Type	References
Glioblastoma multiforme	[14,15,16,17,18]
Head and neck	[19,20,21,22]
Melanoma	[23,24]
Metastatic colorectal carcinoma	[25]

## Data Availability

This study does not report any new data.
